# Supplementary material for: Heparin-based hydrogel scaffolding alters the transcriptomic profile and increases the chemoresistance of MDA-MB-231 triple-negative breast cancer cells
Source: Biomater Sci. 2020 Feb 13;8(10):2786–96. doi: 10.1039/c9bm01481k (PMC7497406; doi:10.1039/c9bm01481k)
Supplement: Supplementary file 2 [file BM-008-C9BM01481K-s002.zip › Supplementary File 4/HP-B hydrogel + EGF (s)vControl/Pathways/my_analysis.Gsea.1545201170987/HALLMARK_KRAS_SIGNALING_DN.html]

Details for gene set HALLMARK\_KRAS\_SIGNALING\_DN[GSEA]

|  || Dataset | expr.class.cls#HYDEGF\_versus\_CONTROL.class.cls#HYDEGF\_versus\_CONTROL\_repos |
| Phenotype | class.cls#HYDEGF\_versus\_CONTROL\_repos |
| Upregulated in class | HYDEGF |
| GeneSet | HALLMARK\_KRAS\_SIGNALING\_DN |
| Enrichment Score (ES) | 0.20570524 |
| Normalized Enrichment Score (NES) | 0.8322446 |
| Nominal p-value | 0.81546575 |
| FDR q-value | 0.83079106 |
| FWER p-Value | 1.0 |
Table: GSEA Results Summary

  

Fig 1: Enrichment plot: HALLMARK\_KRAS\_SIGNALING\_DN      
 Profile of the Running ES Score & Positions of GeneSet Members on the Rank Ordered List

  

| PROBE | DESCRIPTION (from dataset) | GENE SYMBOL | GENE\_TITLE | RANK IN GENE LIST | RANK METRIC SCORE | RUNNING ES | CORE ENRICHMENT || 1 | MSH5 | na |  |  | 59 | 3.172 | 0.0452 | Yes |
| 2 | EGF | na |  |  | 131 | 2.796 | 0.0840 | Yes |
| 3 | EDN1 | na |  |  | 166 | 2.681 | 0.1230 | Yes |
| 4 | TGM1 | na |  |  | 1199 | 1.569 | 0.0929 | Yes |
| 5 | CCNA1 | na |  |  | 1442 | 1.452 | 0.1023 | Yes |
| 6 | SPTBN2 | na |  |  | 1651 | 1.374 | 0.1123 | Yes |
| 7 | SYNPO | na |  |  | 1915 | 1.287 | 0.1181 | Yes |
| 8 | DTNB | na |  |  | 1930 | 1.283 | 0.1369 | Yes |
| 9 | NRIP2 | na |  |  | 2223 | 1.204 | 0.1400 | Yes |
| 10 | KCND1 | na |  |  | 2283 | 1.186 | 0.1549 | Yes |
| 11 | CPEB3 | na |  |  | 2296 | 1.182 | 0.1723 | Yes |
| 12 | DLK2 | na |  |  | 2501 | 1.128 | 0.1788 | Yes |
| 13 | GTF3C5 | na |  |  | 2774 | 1.058 | 0.1806 | Yes |
| 14 | CLSTN3 | na |  |  | 3394 | 0.923 | 0.1623 | Yes |
| 15 | UPK3B | na |  |  | 3978 | 0.815 | 0.1442 | Yes |
| 16 | ATP6V1B1 | na |  |  | 4023 | 0.808 | 0.1542 | Yes |
| 17 | CHRNG | na |  |  | 4052 | 0.804 | 0.1649 | Yes |
| 18 | TFCP2L1 | na |  |  | 4191 | 0.783 | 0.1696 | Yes |
| 19 | SPHK2 | na |  |  | 4289 | 0.768 | 0.1762 | Yes |
| 20 | NR6A1 | na |  |  | 4725 | 0.703 | 0.1642 | Yes |
| 21 | H2AFY2 | na |  |  | 4922 | 0.665 | 0.1640 | Yes |
| 22 | EFHD1 | na |  |  | 4990 | 0.653 | 0.1705 | Yes |
| 23 | SHOX2 | na |  |  | 5053 | 0.645 | 0.1770 | Yes |
| 24 | MX1 | na |  |  | 5059 | 0.644 | 0.1866 | Yes |
| 25 | ABCG4 | na |  |  | 5239 | 0.614 | 0.1865 | Yes |
| 26 | CHST2 | na |  |  | 5270 | 0.610 | 0.1943 | Yes |
| 27 | IDUA | na |  |  | 5557 | 0.570 | 0.1880 | Yes |
| 28 | FGFR3 | na |  |  | 5581 | 0.565 | 0.1954 | Yes |
| 29 | BARD1 | na |  |  | 5616 | 0.559 | 0.2021 | Yes |
| 30 | GAMT | na |  |  | 5843 | 0.525 | 0.1983 | Yes |
| 31 | RSAD2 | na |  |  | 5911 | 0.515 | 0.2026 | Yes |
| 32 | GP1BA | na |  |  | 6003 | 0.502 | 0.2055 | Yes |
| 33 | CELSR2 | na |  |  | 6140 | 0.484 | 0.2057 | Yes |
| 34 | MAST3 | na |  |  | 6347 | 0.458 | 0.2019 | No |
| 35 | STAG3 | na |  |  | 6738 | 0.405 | 0.1876 | No |
| 36 | IFI44L | na |  |  | 6789 | 0.397 | 0.1911 | No |
| 37 | TCF7L1 | na |  |  | 6879 | 0.386 | 0.1923 | No |
| 38 | GPR3 | na |  |  | 7195 | 0.344 | 0.1810 | No |
| 39 | MTHFR | na |  |  | 7280 | 0.332 | 0.1817 | No |
| 40 | RIBC2 | na |  |  | 7479 | 0.307 | 0.1760 | No |
| 41 | ENTPD7 | na |  |  | 7610 | 0.289 | 0.1736 | No |
| 42 | KRT15 | na |  |  | 7721 | 0.276 | 0.1720 | No |
| 43 | HTR1D | na |  |  | 7776 | 0.267 | 0.1733 | No |
| 44 | MFSD6 | na |  |  | 7785 | 0.266 | 0.1769 | No |
| 45 | P2RX6 | na |  |  | 7823 | 0.260 | 0.1789 | No |
| 46 | SGK1 | na |  |  | 8039 | 0.239 | 0.1713 | No |
| 47 | EPHA5 | na |  |  | 8251 | 0.215 | 0.1635 | No |
| 48 | LFNG | na |  |  | 8516 | 0.185 | 0.1525 | No |
| 49 | EDN2 | na |  |  | 8553 | 0.181 | 0.1534 | No |
| 50 | C5 | na |  |  | 8726 | 0.161 | 0.1468 | No |
| 51 | GPR19 | na |  |  | 8981 | 0.124 | 0.1354 | No |
| 52 | ITGB1BP2 | na |  |  | 9261 | 0.094 | 0.1223 | No |
| 53 | CACNA1F | na |  |  | 9517 | 0.072 | 0.1100 | No |
| 54 | PRODH | na |  |  | 9582 | 0.063 | 0.1077 | No |
| 55 | HSD11B2 | na |  |  | 9679 | 0.052 | 0.1034 | No |
| 56 | SKIL | na |  |  | 9738 | 0.045 | 0.1011 | No |
| 57 | THRB | na |  |  | 9801 | 0.035 | 0.0984 | No |
| 58 | SERPINB2 | na |  |  | 9903 | 0.024 | 0.0934 | No |
| 59 | TAS2R4 | na |  |  | 10275 | -0.018 | 0.0743 | No |
| 60 | CAMK1D | na |  |  | 10307 | -0.023 | 0.0730 | No |
| 61 | CDKAL1 | na |  |  | 10638 | -0.058 | 0.0567 | No |
| 62 | SLC29A3 | na |  |  | 10761 | -0.074 | 0.0514 | No |
| 63 | CCDC106 | na |  |  | 11228 | -0.127 | 0.0289 | No |
| 64 | BMPR1B | na |  |  | 11316 | -0.139 | 0.0265 | No |
| 65 | PTPRJ | na |  |  | 11812 | -0.205 | 0.0037 | No |
| 66 | PDE6B | na |  |  | 12109 | -0.234 | -0.0082 | No |
| 67 | SNN | na |  |  | 13639 | -0.432 | -0.0816 | No |
| 68 | SLC16A7 | na |  |  | 13836 | -0.464 | -0.0848 | No |
| 69 | COPZ2 | na |  |  | 13876 | -0.471 | -0.0797 | No |
| 70 | BTG2 | na |  |  | 14115 | -0.493 | -0.0847 | No |
| 71 | GPRC5C | na |  |  | 14408 | -0.539 | -0.0917 | No |
| 72 | MAGIX | na |  |  | 14466 | -0.551 | -0.0863 | No |
| 73 | PDK2 | na |  |  | 15010 | -0.634 | -0.1051 | No |
| 74 | SLC25A23 | na |  |  | 15578 | -0.738 | -0.1235 | No |
| 75 | MYO15A | na |  |  | 15609 | -0.744 | -0.1138 | No |
| 76 | PLAG1 | na |  |  | 16465 | -0.939 | -0.1442 | No |
| 77 | TGFB2 | na |  |  | 16607 | -0.972 | -0.1368 | No |
| 78 | KCNN1 | na |  |  | 16728 | -1.006 | -0.1278 | No |
| 79 | SLC6A14 | na |  |  | 16768 | -1.016 | -0.1144 | No |
| 80 | NR4A2 | na |  |  | 16791 | -1.024 | -0.1000 | No |
| 81 | FGGY | na |  |  | 17265 | -1.168 | -0.1069 | No |
| 82 | ASB7 | na |  |  | 17844 | -1.412 | -0.1157 | No |
| 83 | FAM46C | na |  |  | 18621 | -1.949 | -0.1267 | No |
| 84 | YPEL1 | na |  |  | 18637 | -1.969 | -0.0975 | No |
| 85 | TFF2 | na |  |  | 19049 | -2.740 | -0.0773 | No |
| 86 | PTGFR | na |  |  | 19066 | -2.792 | -0.0357 | No |
| 87 | CYP39A1 | na |  |  | 19068 | -2.796 | 0.0068 | No |
Table: GSEA details [plain text format]

  

Fig 2: HALLMARK\_KRAS\_SIGNALING\_DN      
 Blue-Pink O' Gram in the Space of the Analyzed GeneSet

  

Fig 3: HALLMARK\_KRAS\_SIGNALING\_DN: Random ES distribution      
 Gene set null distribution of ES for **HALLMARK\_KRAS\_SIGNALING\_DN**
